# Proteomic Changes in Patients with LADA-Type Diabetes: Prospects for Diagnostic Biomarkers

**DOI:** 10.3390/ijms27125205

**Published:** 2026-06-09

**Authors:** Adam Osowski, Tomasz Antonowski, Joanna Wojtkiewicz

**Affiliations:** Department of Human Physiology and Pathophysiology, School of Medicine, Collegium Medicum, University of Warmia and Mazury, 10-082 Olsztyn, Poland; t.antonowski@gmail.com

**Keywords:** LADA, cytokines, adiponectin, IFN-γ, CCL2, osteopontin, GPLD1, CXCL 10, hs-CRP

## Abstract

Latent autoimmune diabetes in adults (LADA) is a form of diabetes with clinical and biological features overlapping those of type 1 diabetes mellitus (T1DM) and type 2 diabetes mellitus (T2DM), which may complicate early diagnosis and disease classification. Interest in proteomic and related biomarker profiles in LADA has increased in recent years because these markers may improve understanding of its pathophysiology and support more accurate differentiation from other forms of diabetes. This narrative review provides a structured overview of currently available evidence on selected inflammatory, immunological, and metabolic biomarkers associated with LADA. The reviewed literature includes data on adiponectin, IFN-γ, CCL2/MCP-1, IL-6, GPLD1, and other markers potentially linked to immune dysregulation, low-grade inflammation, and progressive β-cell dysfunction. Several studies suggest that LADA may present an intermediate biomarker profile, with some features overlapping with T1DM and others with T2DM. At the same time, the available evidence remains heterogeneous and is limited mainly by small sample sizes, cross-sectional designs, and incomplete clinical validation. Therefore, although several biomarkers appear promising for further investigation, their current role should be regarded as exploratory rather than established for routine clinical use. Further longitudinal and well-characterized studies are needed to determine whether selected biomarker patterns may eventually contribute to improved identification and stratification of LADA.

## 1. Introduction

Diabetes mellitus remains a major global health challenge, affecting more than 589 million adults aged 20–79 years in 2025, with a projected increase to 853 million by 2050 [1]. Among its heterogeneous forms, Latent Autoimmune Diabetes in Adults (LADA) is of particular clinical interest because it shares features of both type 1 diabetes mellitus (T1DM) and type 2 diabetes mellitus (T2DM), which may delay accurate diagnosis and the initiation of appropriate treatment. LADA is typically characterized by adult onset, the presence of islet autoantibodies such as glutamic acid decarboxylase antibodies, and a slower progression toward insulin dependence than in classical T1DM [2,3,4].

LADA accounts for approximately 2–12% of diabetes cases in adults and is often initially misclassified as T2DM, which may contribute to delayed insulin therapy and suboptimal glycemic control [2,5]. Increasing attention has therefore been directed toward biomarkers that may improve disease recognition and characterization. Among the most frequently discussed candidates are inflammatory, immunological, and metabolic markers, including adiponectin, IFN-γ, CCL2/MCP-1 and IL-6 [6,7]. In 2019, the World Health Organization reclassified LADA as “slowly evolving, immune-mediated diabetes of adults” and categorized it among hybrid forms of diabetes. This reclassification reflects the evolving international consensus regarding the disease’s latent clinical course and its position within the spectrum of autoimmune diabetes [8].

However, the currently available literature remains heterogeneous in terms of study design, sample size, biomarker selection, and analytical methods. Many published reports are exploratory or cross-sectional, and the clinical applicability of the proposed biomarkers has not yet been sufficiently validated. A structured overview of the available evidence may therefore help clarify which observations are currently supported by direct data and which remain preliminary.

**Figure 1:**
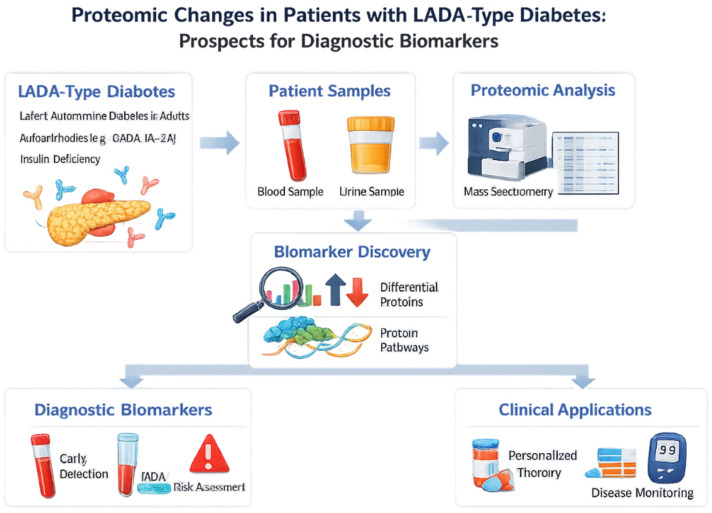
Prospects for diagnostic biomarkers in patients with LADA-type diabetes.

The aim of this review is to provide a structured narrative overview of proteomic and related inflammatory or immunological biomarkers associated with LADA, with particular emphasis on their possible diagnostic and pathophysiological relevance. The review also highlights limitations of the available literature and outlines areas that require further validation in future studies (Figure 1).

## 2. Methodology

This article was designed as a narrative review with a structured approach to literature identification and selection. The review focused on studies addressing proteomic, inflammatory, immunological, and selected metabolic biomarkers associated with latent autoimmune diabetes in adults (LADA), especially in relation to their possible diagnostic and pathophysiological significance.

The literature considered in this review included original research articles, observational studies, case–control studies, cohort studies, and selected review papers relevant to the topic. Priority was given to studies directly involving patients with LADA and reports comparing biomarker profiles between LADA, T1DM, T2DM, and, where applicable, healthy controls.

The available evidence was analyzed thematically and organized into sections concerning clinical characteristics of LADA, inflammatory markers, immunological markers, proteomic findings, angiogenesis-related pathways, and research gaps. Because the literature in this field is still limited and heterogeneous, this work does not represent a formal systematic review or meta-analysis. Instead, it provides a structured narrative synthesis of currently available data.

Particular attention was paid to differences in study design, sample size, and the translational value of the reported biomarkers. When interpreting the literature, emphasis was placed on distinguishing findings supported by direct evidence in LADA from observations extrapolated from T1DM, T2DM, or other autoimmune conditions.

## 3. LADA Characteristics

Latent autoimmune diabetes in adults (LADA) is a form of autoimmune diabetes diagnosed in adulthood that shows clinical, immunological, and metabolic features overlapping those of T1DM and T2DM. Its course is typically slower than that of classical T1DM, which often contributes to delayed recognition and initial misclassification as T2DM. Because of this intermediate presentation, LADA is increasingly regarded as a heterogeneous condition rather than a uniform disease entity, with variability in clinical phenotype, rate of β-cell decline, and metabolic burden [2].

### 3.1. Definition and Diagnostic Criteria

LADA is generally defined by adult onset of diabetes, the presence of diabetes-associated autoantibodies, and the absence of insulin requirement for at least 6 months after diagnosis. In clinical practice, the most characteristic autoantibodies are directed against glutamic acid decarboxylase, although other markers such as IA-2 autoantibodies may also be detected. A major diagnostic difficulty is that patients often present without ketoacidosis and may initially retain sufficient endogenous insulin secretion to resemble T2DM rather than classical autoimmune diabetes. As a result, LADA is frequently diagnosed only after progressive deterioration of glycemic control, declining β-cell function, or confirmation of islet autoimmunity. This diagnostic overlap is one of the main reasons why LADA remains underrecognized in routine clinical practice [3]. The currently used diagnostic criteria are clinically useful, but they do not fully capture the biological heterogeneity of LADA. Patients classified under the same diagnostic label may differ substantially in antibody titers, metabolic profile, insulin resistance, and rate of progression to insulin dependence. For this reason, increasing attention has been paid to additional biomarkers that may improve stratification beyond conventional classification criteria [9].

LADA progresses more slowly than classic type 1 diabetes because the underlying autoimmune destruction of pancreatic beta cells is typically less aggressive. In LADA, autoantibody levels are often lower, and the immune response is less intense, resulting in a gradual decline in insulin-producing cell function rather than the rapid beta-cell loss seen in childhood-onset type 1 diabetes. Adults also generally retain a greater residual beta-cell mass at diagnosis, which allows endogenous insulin production to persist for a longer period. In addition, differences in immune regulation and genetic background may contribute to a milder disease course. While LADA shares features of type 1 diabetes, its overlapping characteristics with type 2 diabetes and the metabolic context of adulthood can further mask early insulin deficiency, contributing to its slower clinical progression [10].

Historically, LADA has sometimes been defined as autoimmune diabetes diagnosed in individuals older than 30 years. However, this cutoff is mainly a research convention rather than a biological boundary. In practice, the age of onset varies considerably. Most patients are diagnosed between the ages of 30 and 50, with a noticeable concentration in the 40 s. This is why some descriptions loosely refer to LADA as “adult-onset autoimmune diabetes.” Still, cases can occur in people in their late 20 s or even earlier 30 s, especially when autoimmune markers such as GAD antibodies are present, and insulin dependence develops gradually. The key point is that age alone is not diagnostic. LADA is identified by its autoimmune nature and slower progression toward insulin dependence, not by a strict age threshold. As a result, modern clinical practice places far more emphasis on antibody testing and metabolic progression than on arbitrary age cutoffs [2].

Similarly to LADA, another form of autoimmune diabetes has been described: Latent Autoimmune Diabetes in Youth (LADY). LADY is an emerging subtype of diabetes in adolescents and young adults that combines features of both type 1 and type 2 diabetes. It is characterized by the presence of pancreatic autoantibodies and a slower progression of β-cell destruction than in classical T1DM. Patients with LADY are often initially diagnosed with type 2 diabetes because they retain endogenous insulin secretion and are not insulin-dependent at onset. However, autoantibodies such as GADA, IA-2A, and ZnT8A indicate autoimmune etiology. Clinical features include varying degrees of insulin resistance, progressive decline in C-peptide levels, and gradual loss of β-cell function, which may delay appropriate insulin therapy. Early recognition is important, as these patients may benefit from earlier insulin treatment and closer metabolic monitoring. Although LADY is not yet universally recognized as a separate diagnostic entity, evidence suggests that autoimmune diabetes in youth exist on a spectrum between T1DM and T2DM. LADY differs from LADA not only by younger age at onset but also by a stronger autoimmune response, more similar to classical T1DM. Patients with LADY typically show higher autoantibody titers and broader immune reactivity against β-cell antigens, leading to faster β-cell decline and earlier insulin dependence [11].

### 3.2. Clinical Characteristics

Clinically, patients with LADA often differ from those with classical T2DM by having a younger age at diagnosis, lower body mass index, a lower degree of insulin resistance, less pronounced dyslipidemia, lower blood pressure, and a lower frequency of metabolic syndrome, although these features usually remain less extreme than in classical T1DM. A personal or family history of autoimmune disease is also more common, supporting the autoimmune background of the condition.

At the same time, the clinical picture is variable. Some individuals present with a phenotype closer to autoimmune diabetes, whereas others show more evident metabolic features that may initially suggest T2DM. This heterogeneity is clinically important because it influences both diagnostic timing and therapeutic decisions. Although many patients initially respond to oral glucose-lowering agents, glycemic control often worsens over time as endogenous insulin secretion declines progressively.

Progression to insulin therapy is generally slower than in T1DM and may occur over several years, often within 5–12 years after diagnosis. This gradual course reflects the less aggressive but still ongoing autoimmune destruction of pancreatic β-cells. Early recognition is therefore relevant not only for diagnostic accuracy but also for treatment planning and monitoring of metabolic deterioration [2,4].

Adiponectin is an adipose-derived hormone that plays a key role in regulating insulin sensitivity and metabolic homeostasis. In general, its circulating levels are inversely associated with insulin resistance, meaning higher adiponectin levels are typically found in individuals with greater insulin sensitivity. In LADA, adiponectin concentrations are commonly higher than in type 2 diabetes, and often closer to those observed in type 1 diabetes. This pattern is primarily explained by the underlying pathophysiology: unlike type 2 diabetes, where insulin resistance and obesity are central features, LADA is driven mainly by autoimmune destruction of pancreatic β-cells, with relatively preserved insulin sensitivity in the early stages of disease. As a result, patients with LADA tend to have a leaner metabolic profile, which is associated with higher adiponectin levels. However, this association is not absolute. Adiponectin levels in LADA can vary depending on factors such as body mass index, residual β-cell function, and disease progression. As insulin deficiency becomes more pronounced and treatment is initiated, metabolic changes may further influence adiponectin concentrations. Overall, while elevated adiponectin may help distinguish a more insulin-sensitive phenotype consistent with LADA compared with type 2 diabetes, it is not a diagnostic biomarker on its own and should be interpreted in the broader clinical and immunological context [12].

In addition, LADA occupies a clinically important position between purely autoimmune and predominantly metabolic diabetes phenotypes. This does not mean that every patient has an equal contribution of both mechanisms; rather, the balance between autoimmunity and metabolic dysfunction appears to differ across individuals. Recognizing this spectrum-like presentation may improve both interpretation of biomarker studies and clinical classification.

### 3.3. Immunological Profile and Proteomics

The immunological profile of LADA supports its classification as autoimmune diabetes, but one with features distinct from classical T1DM. Studies of leukocyte and peripheral blood cell responses have shown altered cytokine and chemokine secretion patterns, suggesting persistent immune activation accompanied by a less abrupt disease course. In particular, increased spontaneous secretion of chemokines such as CCL2/MCP-1, CCL5/RANTES and CCL7/MCP-3 has been described in LADA compared with both T1DM and T2DM. These findings suggest that the inflammatory environment in LADA is characterized by chronic low-grade activation rather than the more fulminant pattern typically associated with rapidly progressive autoimmune diabetes. At the same time, responses to mitogen or antigen stimulation may be lower or otherwise altered, which may indicate differences in immune regulation, activation threshold, or functional responsiveness. Such observations are consistent with the concept that immune dysregulation in LADA is sustained but not identical to that seen in classical T1DM. Proteomic and transcriptomic studies further support this interpretation by showing that LADA is associated with changes in pathways related to inflammation, immune signaling, and cellular regulation. These molecular findings do not yet define a single diagnostic signature, but they do indicate that LADA has a recognizable biological profile that differs from both T1DM and T2DM at the level of immune mediators and cell-response patterns. From a pathophysiological perspective, these observations are important because they provide a mechanistic background for the slower β-cell decline and broader phenotypic diversity observed in LADA [13,14].

### 3.4. Genetic Aspects

The genetic background of LADA also supports its intermediate position between T1DM and T2DM. Available studies indicate that LADA shares susceptibility loci associated with autoimmune diabetes, while also showing overlap with variants linked to metabolic dysfunction or T2DM-related traits. This mixed genetic architecture is consistent with the clinical and immunological heterogeneity observed in affected patients. The HLA region remains highly relevant, but the frequency of high-risk HLA alleles in LADA is generally lower than in classical T1DM. This may partly explain the slower progression of β-cell dysfunction and the less acute clinical presentation at diagnosis. At the same time, the presence of autoimmune susceptibility alleles confirms that LADA should not be viewed simply as T2DM with autoantibodies, but rather as a distinct form of diabetes with a specific immunogenetic background. Additional loci further illustrate this complexity. Variants involving genes such as INS and PTPN22, which are strongly associated with T1DM, have also been identified in LADA, although often with lower intensity or frequency than in classical T1DM. Conversely, TCF7L2 and other loci associated with T2DM may also contribute, particularly in individuals with a stronger metabolic phenotype. This overlap suggests that the relative contribution of immune-mediated and metabolic mechanisms may differ among patients and may influence disease progression. The genetic heterogeneity of LADA has important clinical implications. It may partly explain differences in age at onset, metabolic profile, residual β-cell function, and timing of insulin dependence among individuals classified within the same diagnostic category. Therefore, genetic findings support the view that LADA is best understood as a spectrum of adult-onset autoimmune diabetes with variable metabolic contribution, rather than as a single, uniform subtype [2,15].

#### 3.4.1. HLA Risk

LADA exhibits a lower frequency of high-risk HLA alleles (e.g., DR3/DR4) than classical T1DM. These HLA variants are strongly associated with rapid autoimmune β-cell destruction in type 1 diabetes. Their lower prevalence in LADA partly explains the slower progression of the disease and the milder metabolic presentation at diagnosis. Consequently, patients with LADA rarely present with ketoacidosis at disease onset and often maintain residual insulin secretion for several years after diagnosis. This genetic pattern further supports the concept of LADA as an intermediate form of autoimmune diabetes, positioned between type 1 and type 2 diabetes in both pathophysiology and clinical course. In addition, the heterogeneity of HLA risk profiles in LADA suggests a more complex interaction between genetic susceptibility and environmental triggers. This may contribute to variability in the rate of β-cell decline among individuals. Furthermore, the presence of protective or lower-risk alleles could modulate the intensity of the autoimmune response, leading to a less aggressive disease phenotype. Understanding these genetic differences is important for improving diagnostic classification and may eventually guide more personalized therapeutic approaches [2,15].

Recent evidence further highlights the specific role of the HLA-C–KIR axis in the pathogenesis of LADA. Interactions between HLA-C molecules and killer cell immunoglobulin-like receptors (KIRs) expressed on natural killer cells appear to regulate immune activation and autoimmune responses targeting pancreatic β-cells. This immunogenetic interplay may contribute to the intermediate phenotype of LADA, linking characteristics of both classical autoimmune diabetes and type 2 diabetes, while also influencing disease susceptibility and progression [16].

#### 3.4.2. Other Loci

In individuals with LADA, alleles of the insulin gene (INS) and protein tyrosine phosphatase non-receptor type 22 (PTPN22), typical for T1DM, are present but at moderate intensity [14]. The lack of dominance of obesity-related genes (like TCF7L2 in T2DM) underscores the autoimmune basis, although TCF7L2 is elevated in obese subgroups. This intermediate genetic profile reflects the nature of LADA, combining features of both type 1 and type 2 diabetes at the molecular level. Additionally, the presence of immune-related susceptibility loci supports the role of chronic, low-grade autoimmune activity in the progressive destruction of pancreatic β-cells. Compared to classic T1DM, the slower tempo of β-cell loss in LADA may be influenced by weaker genetic predisposition and environmental modifiers. These findings highlight the heterogeneity of LADA and suggest that both genetic background and metabolic factors contribute to its clinical manifestation and progression [15,17]. The frequency of individual alleles is presented in Table 1.

### 3.5. Proteomic Changes in LADA

Proteomic changes in LADA reflect the intermediate nature of this form of diabetes, combining type 1 (T1DM) autoimmune features with type 2 (T2DM) elements of metabolic dysfunction and inflammation. Studies have shown alterations in proteins involved in immune signaling, inflammatory pathways, oxidative stress, and metabolic regulation [3]. These proteomic signatures may provide valuable insights into the pathophysiological mechanisms underlying LADA and may also serve as potential biomarkers for earlier and more accurate diagnosis. Understanding these molecular patterns may help differentiate LADA from other forms of diabetes and support the development of more personalized therapeutic strategies. Furthermore, proteomic profiling may enable the identification of disease subtypes and progression patterns, allowing clinicians to predict the rate of beta-cell decline and treatment response. Emerging technologies in mass spectrometry and bioinformatics are also improving the sensitivity and reproducibility of proteomic analyses, increasing their clinical relevance. In the future, integrating proteomic data with genetic, metabolomic, and clinical information may contribute to precision medicine approaches for patients with LADA [7,18].

It should be emphasized that current evidence regarding JAK/STAT inhibition in autoimmune diabetes is derived mainly from studies conducted in patients with classical T1DM. The landmark BANDIT trial and the ongoing JAKPOT T1D study predominantly include individuals aged 10–30 and 12–35 years, respectively. Although the immunomodulatory mechanisms of JAK/STAT pathway inhibition appear highly relevant to the pathophysiology of LADA, dedicated large-scale randomized controlled trials in this population are still lacking. As a result, therapeutic management of LADA continues to rely primarily on expert consensus and individualized assessment of residual β-cell function, particularly C-peptide levels, rather than on evidence from disease-modifying intervention studies specifically designed for LADA [19].

#### 3.5.1. Key Proteomic Markers of LADA

Plasma and leukocyte proteomic studies highlight several characteristic biomarkers. The level of glycosylphosphatidylinositol phospholipase D glycoprotein (GPLD1) is elevated in LADA, similar to that observed in T1DM and significantly higher than in T2DM, making it a potential tool for identifying early LADA, even before the appearance of high titers of classical autoantigens [6,17]. Adiponectin shows an intermediate profile in LADA—higher than in T2DM (with low adiponectin levels associated with insulin resistance), but lower or comparable to T1DM, correlating with less severe metabolic syndrome in this patient group [9,12]. Chemokines CCL2/MCP-1 and CCL5/RANTES are spontaneously secreted to a greater degree by leukocytes in LADA than in T1DM and T2DM, indicating a low-grade inflammatory state with a mixed profile, however, stimulation with antigens or mitogens reduces their response, which may explain the slower disease progression [13,20].

The diagnostic utility of GPLD1 is currently being evaluated in combination with other protein biomarkers. Recent studies have demonstrated that multi-biomarker panels incorporating proteins such as VWF and CAT achieve high predictive accuracy for pancreatic inflammatory severity, with reported AUC values of approximately 0.903. These findings suggest that GPLD1 may provide the greatest clinical value when incorporated into a broader proteomic signature rather than used as a standalone biomarker [21].

It is important to note that circulating VEGF levels may be significantly influenced by the sample collection and processing method. In particular, citrate plasma has been shown to be unsuitable for reliable VEGF assessment because of the artificial release of VEGF from platelets during sample processing. This methodological limitation may partly explain the inconsistent findings reported in the literature regarding the role of VEGF in endothelial dysfunction [22].

#### 3.5.2. Immunological Profile and Transcriptomics

**Figure 2:**
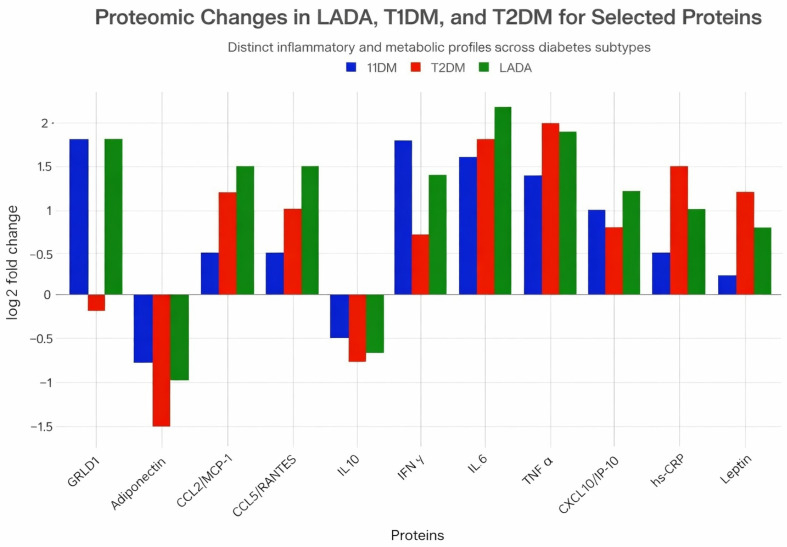
Proteomic changes in LADA, T1DM and T2DM. Changes shown as log2 FC (base-2 log of FC, where FC = fold change—relative expression fold change vs. healthy controls). log2 FC > 0: upregulation (e.g., +1.8 = FC ≈3.5-fold increase); log2 FC < 0: downregulation (e.g., −1.0 = FC 0.5–2-fold decrease); ≈0: no change. Approximate values from studies [9,13,26,27].

LADA is characterized by a mixed immunological profile with elevated secretion of IL-10 (anti-inflammatory) and IFN-γ (pro-inflammatory) following stimulation with beta-cell antigens, emphasizing the autoimmune-inflammatory dualism [23,24]. Transcriptomics of peripheral blood mononuclear cells (PBMC) reveal suppression of the NF-κB pathway as well as increased activity of pathways related to IL-6, TNF-α, and CXCL10/IP-10, integrating proteomic changes with transcriptional regulation [24]. Low systemic inflammation markers, such as hs-CRP or leptin, complement this picture, enabling proteomic-immunological differentiation of early LADA from T2DM with greater precision than autoantibodies alone (GADA) [9,20,25]. Qin W. et al. [26] show elevated GPLD1 in LADA like T1DM, differing from T2DM, while Ooms M. et al. [13] confirm higher secretion of CCL2 and CCL5 in LADA. The following Figure 2 and Table 2 present proteomic changes in the main types of diabetes based on literature data.

The level of glutamic acid decarboxylase autoantibodies (GADA) plays an important role in determining the clinical phenotype and rate of disease progression in LADA. GADA titers are considered not only diagnostic markers of autoimmune diabetes, but also prognostic indicators reflecting the intensity of the autoimmune response. Patients with high GADA titers typically present with features more similar to T1DM, including lower endogenous insulin secretion, faster decline in C-peptide levels, and earlier progression to insulin dependence. High-titer GADA positivity is also more frequently associated with multiple pancreatic autoantibodies, suggesting broader immune activation against β-cell antigens. In contrast, patients with low GADA titers often exhibit metabolic characteristics more typical of T2DM, such as obesity, insulin resistance, and slower progression toward β-cell failure. Quantitative assessment of GADA may therefore provide clinically relevant prognostic information and help guide individualized therapeutic strategies, including the timing of insulin initiation and the intensity of metabolic monitoring [25].

## 4. LADA Inflammatory Markers

Available studies suggest that LADA is associated with a low-grade inflammatory profile that differs from both classical T1DM and T2DM, although the boundaries between these phenotypes remain imperfectly defined. Reported changes include altered levels of CCL2/MCP-1, IL-6, CRP, TNF-α, and other inflammatory mediators, supporting the view that inflammatory activation contributes to the pathophysiology of LADA in parallel with autoimmune β-cell injury [9,13,26]. At the same time, the available evidence is derived mainly from cross-sectional and exploratory studies, which limits the strength of causal and clinical inferences. One of the most consistent observations concerns chemokine dysregulation. Increased spontaneous secretion of CCL2/MCP-1 and CCL5/RANTES by peripheral blood mononuclear cells has been reported in LADA compared with both T1DM and T2DM [13]. These findings are of particular interest because they suggest persistent baseline immune activation rather than only an exaggerated response to acute stimulation. However, the interpretation is not straightforward, since stimulated responses may be lower than expected, indicating that immune activation in LADA may coexist with altered regulatory or functional responsiveness. This pattern may help explain why LADA follows a slower clinical course than classical T1DM despite ongoing autoimmune processes. Chemokines such as CCL2/MCP-1 and CCL5/RANTES are involved in recruitment of monocytes and lymphocytes and may therefore contribute to sustained inflammatory activity within the pancreatic microenvironment. Nevertheless, the available data do not yet establish whether these mediators are specific biomarkers of LADA or rather reflect broader inflammatory processes shared with other forms of diabetes and chronic metabolic dysfunction.

Other inflammatory mediators have also been implicated in LADA [27]. Increased levels or altered responses of IL-6, TNF-α, CRP, IL-1β, IFN-γ, and M-CSF have been described, further supporting the presence of a persistent but relatively moderate inflammatory state. Compared with classical T1DM, this inflammatory pattern appears less abrupt, while in comparison with T2DM it may be more closely linked to immune-mediated β-cell damage. Such observations support the concept that inflammation in LADA is not merely secondary to metabolic imbalance, although the relative contribution of autoimmunity and metabolic stress likely varies across patients.

Particularly relevant are data showing that patients with LADA may exhibit elevated IL-10 production in response to insulin or GAD65 stimulation together with increased pro-inflammatory cytokine responses. This coexistence of pro-inflammatory and regulatory signals suggests that the inflammatory milieu in LADA is more complex than a simple activated-versus-non-activated model. It may instead reflect a state of chronic immune disequilibrium in which inflammatory and compensatory mechanisms operate simultaneously.

In diabetes, MAP kinase (MAPK) signaling pathways are often overactivated due to chronic hyperglycemia, oxidative stress, and systemic inflammation. This sustained activation contributes to insulin resistance and diabetic complications. Notably, JNK-mediated serine phosphorylation of insulin receptor substrate-1 (IRS-1) impairs insulin signaling, while p38 MAPK and ERK1/2 drive pro-inflammatory gene expression, maintaining chronic low-grade inflammation in metabolic tissues such as liver, skeletal muscle, and adipose tissue. Together, these pathways integrate metabolic and inflammatory stress signals, reinforcing insulin resistance and tissue dysfunction. Evidence also suggests that MAPK activation contributes to pancreatic β-cell dysfunction through pro-apoptotic signaling and reduced adaptive capacity under metabolic stress, highlighting these pathways as potential therapeutic targets in diabetes [28].

In recent years, increasing attention has focused on inflammatory cytokines in the pathogenesis of LADA, particularly tumor necrosis factor-alpha (TNF-α) and interleukin-1 beta (IL-1β). These mediators drive chronic inflammation and pancreatic β-cell damage. TNF-α, produced mainly by macrophages, T lymphocytes, and adipose tissue, contributes to persistent low-grade inflammation in LADA. It impairs insulin secretion, promotes β-cell apoptosis, and may increase insulin resistance. Because LADA has both autoimmune and metabolic features, TNF-α may link type 1–like autoimmunity with the inflammatory profile of type 2 diabetes. Elevated levels are also associated with poorer glycemic control and faster C-peptide decline [29]. IL-1β is another key cytokine involved in islet damage. It promotes oxidative stress and nitric oxide production in β-cells, reducing insulin secretion and accelerating cell death. It may act early in the disease process before full insulin dependence develops [30]. Overall, the inflammatory profile of LADA appears intermediate between type 1 and type 2 diabetes, which may explain its slower progression. TNF-α and IL-1β often act synergistically via NF-κB activation, increased immune-cell recruitment, and oxidative stress, thereby accelerating β-cell dysfunction. These cytokines are being studied as potential biomarkers and therapeutic targets in autoimmune diabetes [29,30].

From a diagnostic perspective, low-grade inflammatory markers may help distinguish LADA from other adult-onset diabetes phenotypes, especially when interpreted together with autoantibodies and clinical features. However, their practical value remains uncertain. Most studies involve relatively small groups, use different analytical methods, and evaluate different marker panels, which makes direct comparison difficult and reduces immediate clinical applicability.

Overall, inflammatory marker studies support the view that LADA is accompanied by persistent immune and inflammatory activation, but current evidence is not sufficient to define a validated inflammatory signature for routine diagnosis. At present, these markers should be regarded primarily as biologically informative and potentially useful for future stratification studies rather than as established standalone clinical tools [9].

## 5. LADA Immunological Markers

The immunological profile of LADA is defined primarily by the presence of islet autoimmunity, which distinguishes this form of diabetes from classical T2DM and places it within the spectrum of autoimmune diabetes. Among the currently used markers, antibodies against glutamic acid decarboxylase remain the most characteristic and clinically relevant, as their presence supports the diagnosis of LADA in adults with a non-insulin-dependent onset of diabetes. However, the immunological profile of LADA extends beyond autoantibody positivity alone and includes broader changes in cytokine signaling, B-cell regulation, and cellular immune responses. A central element of this profile is the coexistence of autoimmune activity with evidence of partial immune regulation rather than fulminant immune destruction. In this respect, LADA differs from classical T1DM, in which β-cell loss is often more rapid, while also differing from T2DM, where autoimmune markers are absent. This intermediate pattern may help explain the slower clinical course of LADA and the prolonged period before insulin dependence develops. At the same time, available studies suggest substantial heterogeneity, indicating that the intensity and composition of the autoimmune response may vary considerably among patients [31].

Several studies have reported altered cytokine responses after stimulation with β-cell autoantigens [32,33,34]. Increased secretion of IFN-γ and IL-2 supports the involvement of Th1-associated immune pathways, while elevated IL-17A suggests participation of Th17-related responses in the chronic autoimmune process. These observations are consistent with ongoing adaptive immune activation directed against pancreatic β-cells, although the magnitude of this response appears less aggressive than in classical T1DM. Importantly, pro-inflammatory immune activation in LADA may coexist with signals suggestive of immunoregulation. Increased IL-10 responses have been described following stimulation with insulin or GAD65-related antigens, which may reflect compensatory mechanisms that partially restrain immune-mediated tissue injury. In addition, a higher proportion of regulatory B cells and IL-35-producing antigen-presenting cells has been reported in some studies, supporting the concept that the immune profile of LADA includes not only pathogenic but also counter-regulatory elements. This balance between autoimmune activation and partial regulation may be one of the factors underlying the slower tempo of β-cell decline in LADA.

BAFF/TNFSF13B is another molecule of potential interest because of its role in B-cell survival and antibody-related immune responses. Increased BAFF-related activity may support the persistence of autoreactive B-cell populations and sustained autoantibody production, thereby contributing to chronic islet-directed autoimmunity. However, the available evidence remains limited, and BAFF should currently be interpreted as a biologically plausible rather than clinically validated marker in LADA. CXCL10/IP-10 and related chemokine pathways also appear relevant to the immunological milieu of LADA [35,36,37]. CXCL10 is involved in recruitment of activated T lymphocytes and has been linked to autoimmune diabetes more broadly. In LADA, elevated spontaneous levels have been reported, although responses after stimulation may be less clearly distinct from those observed in T1DM or T2DM. This suggests that chemokine-related immune recruitment may contribute to disease activity, but its diagnostic specificity remains uncertain.

Another molecule, GLP-1 receptor agonists (GLP-1RAs), such as semaglutide, may provide additional benefits in patients with LADA by promoting favorable changes in adipose tissue distribution, particularly through the reduction in visceral fat accumulation. Furthermore, these agents have been shown to significantly increase serum adiponectin levels, which may contribute to the attenuation of both metabolic stress and vascular injury. Consequently, GLP-1RAs may exert a dual protective effect by improving metabolic control while simultaneously reducing the risk of diabetes-related vascular complications in LADA [38].

Taken together, currently available studies indicate that LADA is characterized by a complex immunological profile combining autoantibody positivity, T-cell activation, B-cell-related signaling, and partial immune regulation. These findings are biologically informative and support the classification of LADA as a distinct form of adult-onset autoimmune diabetes. Nevertheless, the literature remains limited by small cohorts, heterogeneous study designs, and inconsistent marker panels, which makes direct comparison difficult and currently prevents translation of these findings into validated immunological tools for routine clinical practice.

## 6. LADA Angiogenic Markers

Direct evidence linking angiogenic markers such as VEGF and VCAM-1 specifically to LADA remains limited. Although these molecules have been studied in other autoimmune diseases and in type 2 diabetes, their role in LADA has not been clearly established. Therefore, their discussion in the context of LADA should be regarded as exploratory rather than conclusive.

### 6.1. VEGF in LADA

No direct studies have clearly established the role of VEGF in LADA. Available evidence derives mainly from other autoimmune disorders and from broader diabetes research, where VEGF has been associated with inflammation, angiogenesis, and microvascular complications [39,40]. In the context of LADA, VEGF may be considered a candidate for future investigation, but current evidence is insufficient to support its use as a disease-specific biomarker.

### 6.2. VCAM-1 in LADA

VCAM-1 has been associated with endothelial dysfunction and microvascular complications in diabetes, particularly in T2DM [41,42]. However, direct data in LADA are lacking. Given the overlap between autoimmune and metabolic features in LADA, VCAM-1 may be of potential interest in future studies, especially in relation to vascular complications, but its relevance in this population remains uncertain.

### 6.3. Implications and Research Gaps

Taken together, the limited evidence does not allow firm conclusions regarding the role of VEGF or VCAM-1 in LADA [3,42,43,44,45,46,47,48,49,50,51]. At present, these markers should be viewed as hypothesis-generating rather than clinically applicable. Future studies should assess their potential relevance in well-characterized LADA cohorts and determine whether they provide information beyond established inflammatory and immunological markers.

## 7. Conclusions

Available evidence suggests that LADA is associated with a distinct but heterogeneous biomarker profile combining inflammatory, immunological, and metabolic features that overlap partly with both T1DM and T2DM. Among the markers discussed in the current literature, adiponectin, IFN-γ, CCL2/MCP-1 and IL-6 appear to be of particular interest for further study, although the strength of evidence remains uneven across studies.

At present, most proposed biomarkers should be regarded as promising research candidates rather than clinically validated diagnostic tools. The available studies are limited by heterogeneity, relatively small sample sizes, and the predominance of cross-sectional designs. Therefore, the current literature supports cautious interpretation rather than immediate clinical implementation.

Further research should focus on larger and better-characterized cohorts, longitudinal study designs, and validation of findings across independent populations. Such studies may help determine whether selected biomarker panels could eventually support improved classification and earlier recognition of LADA in clinical practice.

## Figures and Tables

**Table 1:** Genetic comparison of allele frequency in diabetes.

Gene/Locus	LADA	T1DM	T2DM	References
HLA DR3/DR4	low	high	neutral	[2,14]
INS VNTR	present, moderate	high	low	[14]
PTPN22	present	high	neutral/ slightly increased	[14]
TCF7L2	low/ increased in obesity	neutral	high	[14,15]

**Table 2:** Proteomic changes in LADA, T1DM and T2DM. Changes shown as log2 FC (base-2 log of FC, where FC = fold change—relative expression fold change vs. healthy controls). log2 FC > 0: upregulation (e.g., +1.8 = FC ≈3.5-fold increase); log2 FC < 0: downregulation (e.g., −1.0 = FC 0.5–2-fold decrease); ≈0: no change. Approximate values from studies [9,13,25,27].

Protein	T1DM	T2DM	LADA	References
GPLD1	+1.8	−0.2	+1.8	[26]
Adiponectin	−0.8	−1.5	−1.0	[27]
CCL2/MCP-1	+0.5	+1.2	+1.5	[13]
CCL5/RANTES	+0.5	+1.0	+1.5	[13]
IL-10	−0.5	−0.8	−0.7	[9]
IFN-γ	+1.8	+0.7	+1.4	[13]
IL-6	+1.6	+1.8	+2.2	[9]
TNF-α	+1.4	+2.0	+1.9	[9]
CXCL10/IP-10	+1.0	+0.8	+1.2	[13]
hs-CRP	+0.5	+1.5	+1.0	[9]
Leptin	+0.2	+1.2	+0.8	[27]

## Data Availability

No new data were created or analyzed in this study. Data sharing is not applicable to this article.
